# Resveratrol inhibits epithelial-mesenchymal transition of retinal pigment epithelium and development of proliferative vitreoretinopathy

**DOI:** 10.1038/srep16386

**Published:** 2015-11-10

**Authors:** Keijiro Ishikawa, Shikun He, Hiroto Terasaki, Hossein Nazari, Huiming Zhang, Christine Spee, Ram Kannan, David R Hinton

**Affiliations:** 1 Arnold and Mabel Beckman Macular Research Center, Doheny Eye Institute, Los Angeles, CA, USA; 2 Departments of Ophthalmology, Keck School of Medicine of the University of Southern California, Los Angeles, CA, USA; 3 Pathology, Keck School of Medicine of the University of Southern California, Los Angeles, CA, USA

## Abstract

Proliferative vitreoretinopathy (PVR) is a serious complication of retinal detachment and ocular trauma, and its recurrence may lead to irreversible vision loss. Epithelial to mesenchymal transition (EMT) of retinal pigment epithelial (RPE) cells is a critical step in the pathogenesis of PVR, which is characterized by fibrotic membrane formation and traction retinal detachment. In this study, we investigated the potential impact of resveratrol (RESV) on EMT and the fibrotic process in cultured RPE cells and further examined the preventive effect of RESV on PVR development using a rabbit model of PVR. We found that RESV induces mesenchymal to epithelial transition (MET) and inhibits transforming growth factor-β2(TGF-β2)-induced EMT of RPE cells by deacetylating SMAD4. The effect of RESV on MET was dependent on sirtuin1 activation. RESV suppressed proliferation, migration and fibronectin synthesis induced by platelet-derived growth factor-BB or TGF-β2. *In vivo*, RESV inhibited the progression of experimental PVR in rabbit eyes. Histological findings showed that RESV reduced fibrotic membrane formation and decreased α-SMA expression in the epiretinal membranes. These results suggest the potential use of RESV as a therapeutic agent to prevent the development of PVR by targeting EMT of RPE.

Proliferative vitreoretinopathy (PVR) can occur in eyes after rhegmatogenous retinal detachment or after major ocular trauma and its surgical repair. The hallmark of PVR is the formation of subretinal, epiretinal and intravitreal fibrotic membranes that can lead to traction retinal detachment^1^. Surgical removal of the fibrotic membranes with retinal detachment repair is the primary treatment for PVR. Despite recent progress in surgical techniques, recurrent detachment can lead to irreversible damage and a poor visual outcome^2^.

The pathology of the fibrotic PVR membrane represents an excessive wound healing response characterized by cellular proliferation and migration, with extracellular matrix (ECM) production and remodeling^3^. A major cellular component in the fibrotic membrane is retinal pigment epithelial (RPE) cells transdifferentiated into myofibroblastic cells through epithelial to mesenchymal transition (EMT)^4^.

In the physiological condition, RPE are arranged as a monolayer of highly polarized cells located between the neural retina and the choroid^5^. During retinal detachment, RPE cells are dislodged from their monolayer into the vitreous cavity or subretinal space with loss of RPE cell-cell contact. There they undergo EMT to a fibrotic phenotype and play a critical role in the development of PVR^6^. Previous studies have indicated that several growth factors and cytokines are involved in EMT of RPE cells, i.e. platelet-derived growth factor (PDGF), transforming growth factor (TGF)-β fibroblast growth factor, epidermal growth factor, and tumor necrosis factor-α^7^,^8^. However, the precise molecular mechanisms remain unclear and an effective therapeutic approach to PVR by targeting EMT of RPE has not yet been developed.

Resveratrol (RESV, trans-3,4,5-trihydroxystilbene) is a polyphenol phytoalexin that is synthesized in a number of plants, including berries, peanuts, and red grapes^9^. RESV has been shown to play anti-oxidant, anti-inflammatory and anti-proliferative roles having beneficial effects in reducing risk of diabetes, heart disease and cancer^10^,^11^. The effects of RESV are mediated through sirtuin 1 (SIRT1) activation, which in turn modulates cellular function by deacetylation of transcription factors and other proteins^12^,^13^.

Previous reports showed that RESV could inhibit the progression of retinal diseases such as age-related macular degeneration and diabetic retinopathy by acting as an anti-angiogenic agent and providing cell protection against stimuli such as oxidative stress, inflammation and light-induced retinal degeneration^14^,^15^,^16^,^17^ Furthermore, RESV has been shown to prevent fibrosis development through mesenchymal-epithelial transition (MET) induction in various cell types: e.g. lung and renal epithelial cells^18^,^19^,^20^. However, the link between RESV, EMT of RPE cells and PVR has not been hitherto studied.

Herein, we investigated the impact of RESV on EMT and the fibrotic process in cultured RPE cells; we further examined the potential of RESV as a therapeutic agent using an *in vivo* model of PVR.

## Results

### Resveratrol inhibits EMT of RPE cells through deacetylation of SMAD4.

**Figure 1:**
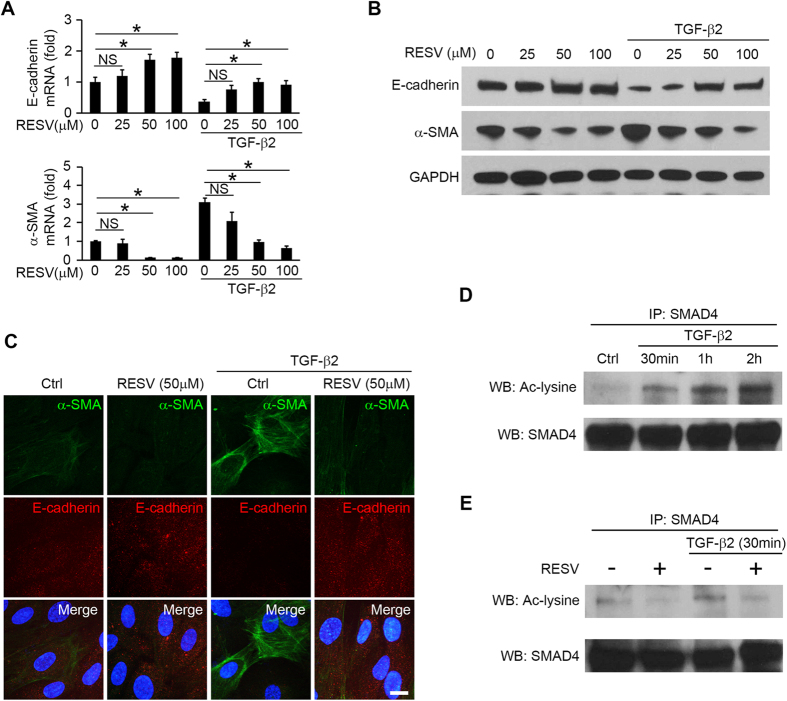
RESV induces MET and inhibits TGF-β2 induced EMT by deacetylating SMAD4. After 1 h pretreatment of RESV at 0, 25, 50 and 100 μM, the cells were stimulated with or without TGF-β2 at 10 ng/ml. (**A**) mRNA expression of E-cadherin and α-SMA are shown as relative fold to control normalized to GAPDH. NS, not significant. *P < 0.05. Data are presented as mean ± SEM. *n* = 4/group. (**B**) Western blot analysis of E-cadherin, α-SMA and the housekeeping protein GAPDH in the cell lysates of RPE cells. An expanded image of the western blot of E-cadherin and its molecular size are shown in Supplementary Figure 6. (**C**) Triple immunofluorescence staining for α-SMA and E-cadherin in RPE cells. Nuclei are stained blue. Scale bar: 10 μm. (**D**) Western blot (WB) analysis with acetyl-lysine (Ac-lysine) and SMAD4 in anti-SMAD4 immunoprecipitates (IP) shows a time course of TGF-β2 (10 ng/ml)-induced SMAD4 acetylation. (**E**) After 1 h pretreatment with RESV at 50 μM, the cells were stimulated with or without TGF-β2 at 10 ng/ml for 30 min. WB analysis of Ac-lysine and SMAD4 in anti-SMAD4 IP from the cells.

To investigate whether RESV can change EMT in RPE cells, we examined changes in expression of E-cadherin (epithelial marker), zonula occludens-1 (ZO-1, epithelial marker) and α-smooth muscle actin (α-SMA, mesenchymal marker) in RPE cells treated with RESV. TGF-β2 is the predominant TGF-β isoform in the posterior eye and a crucial inducer of EMT in RPE cells^21^. Treatment with TGF-β2 (10 ng/ml for 48 h) significantly reduced expression of E-cadherin and ZO-1, and increased expression of α-SMA at the mRNA and protein level, whereas treatment with RESV at 50 μM and 100 μM significantly increased E-cadherin, and ZO-1, and decreased α-SMA protein and mRNA levels. The TGF-β2-induced decrease of E-cadherin and ZO-1, and increase of α-SMA could be inhibited by RESV at 50 μM and 100 μM (Fig. 1A,B). The RT-qPCR analyses for mRNA expression were normalized using two reference genes (GAPDH; Fig. 1A; β-actin, supplementary Figure 1A and supplementary Figure 2). Immunofluorescence staining validated the effect of RESV on the expression of E-cadherin and α-SMA, with or without TGF-β2 treatment (Fig. 1C), suggesting that RESV induces MET (increased E-cadherin and decreased α-SMA) and could inhibit TGF-β2-induced EMT. To examine whether TGF-β2 can induce acetylation of SMAD4, we immunoprecipitated SMAD4 from RPE treated with TGF-β2 and performed western blots with antibodies against SMAD4 or acetyl lysine. Significant SMAD4 acetylation was seen at 30 min, 1 h and 2 h after treatment with TGF-β2 (Fig. 1D, supplementary Figure 3A). RESV inhibited SMAD4 acetylation with or without TGF-β2 treatment for 30 min (Fig. 1E, supplementary Figure 3B), suggesting that RESV induces MET and inhibits TGF-β2 induced EMT of RPE cells by deacetylation of SMAD4.

### SIRT1 mediates a facilitatory role of RESV on MET

**Figure 2:**
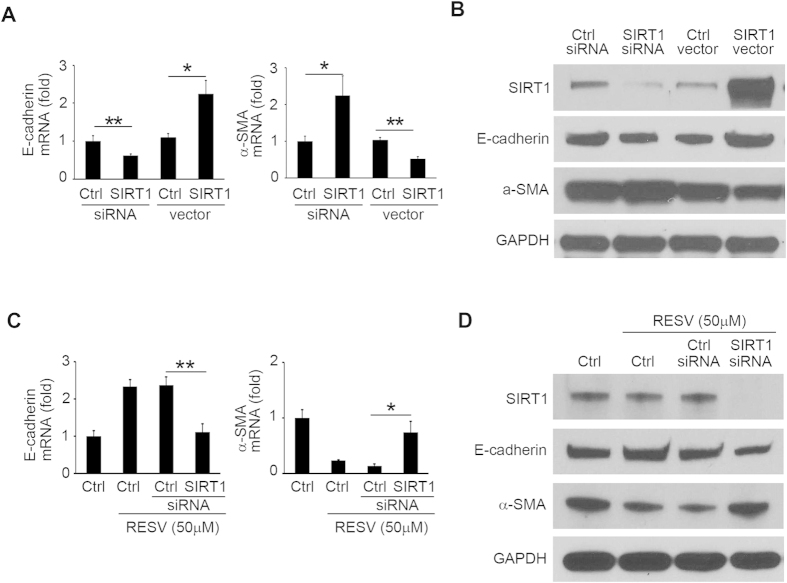
Facilitatory role of RESV on MET is dependent on SIRT1. After transfection with control siRNA, SIRT1 siRNA, empty vector, and SIRT1-encoding vector, RPE cells were incubated for 48 h. (**A**) mRNA expression of SIRT1, E-cadherin and α-SMA are shown as relative fold to control siRNA normalized to GAPDH. **P < 0.01. *P < 0.05. Data are presented as mean ± SEM. *n* = 4/group. (**B**) Western blot analysis of SIRT1, E-cadherin, α-SMA and GAPDH in the cell lysates of RPE cells. An expanded image of the western blot of E-cadherin is shown in Supplementary Figure 6. (**C**) After transfection with control siRNA and SIRT1 siRNA, the cells were incubated with RESV at 50 μM for 48 h. mRNA expression of SIRT1, E-cadherin and α-SMA are shown as relative fold to control siRNA normalized to GAPDH. **P < 0.01. *P < 0.05. Data are presented as mean ± SEM. *n* = 4/group. (**D**) Western blot analysis of SIRT1, E-cadherin, α-SMA and GAPDH in the cell lysates of RPE cells. An expanded image of the western blot of E-cadherin is shown in Supplementary Figure 6.

To test whether modulation of SIRT1 expression can regulate EMT markers, we examined the changes in expression of E-cadherin and α-SMA in RPE cells transfected with SIRT1 siRNA and SIRT1-encoding vector. Silencing of SIRT1 by siRNA significantly decreased E-cadherin and increased α-SMA, while overexpression of SIRT1 by DNA vector increased E-cadherin and decreased α-SMA in protein and mRNA levels (Fig. 2A,B; see also supplementary Figure 4A,B). Next, to study whether RESV facilitates MET through SIRT1, we examined the effect of SIRT1 silencing on RESV-induced expression change of EMT markers in RPE cells. After silencing of SIRT1, RESV did not alter expression of E-cadherin and α-SMA in mRNA (Fig. 2A,C and supplementary Figure 4A,C) and protein levels (Fig. 2B,D; see also supplementary Figure 4B–D). These results suggest that SIRT1 is a crucial mediator in the facilitatory effect of RESV on MET of RPE cells.

### RESV inhibits cell proliferation, migration and fibronectin synthesis

**Figure 3:**
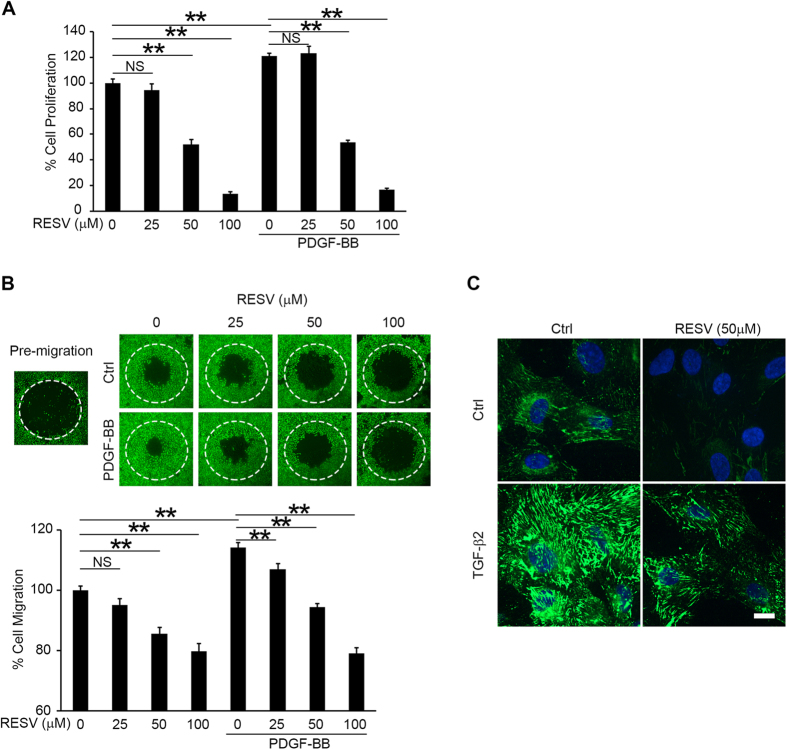
RESV inhibits cell proliferation, migration and fibronectin synthesis. After 1 h pretreatment with RESV at 0, 25, 50 and 100 μM, the cells were stimulated with or without PDGF-BB at 50 ng/ml for 48 h. (**A**) BrdU incorporations were measured to assay proliferation. NS, not significant. **P < 0.01. Data are presented as mean ± SEM. *n* = 4/group. (**B**) In the migration assay, the areas of the cells (stained by calcein AM) that migrated into the detection zone (white dotted circle) were measured. NS, not significant. **P < 0.01. Data are presented as mean ± SEM. *n* = 4/group. (**C**) Immunofluorescence staining of fibronectin in RPE cells with or without 10 ng/ml TGF-β2 stimulation for 12 h. Scale bar: 10 μm.

EMT of RPE cells is an initial step in fibrotic processes such as cell proliferation, migration, and ECM remodeling in the pathogenesis of PVR^3^. PDGF-BB is an important PVR-driving growth factor that is expressed in PVR fibrotic membranes and associated with proliferation and migration of RPE cells^22^. Fibronectin, an adhesive glycoprotein, is a critical ECM component that provides a provisional matrix for migration of RPE cells^23^,^24^. We next tested the effect of RESV on cell proliferation, migration and fibronectin synthesis in RPE cells. PDGF-BB significantly promoted cell proliferation, as shown by increased BrdU incorporation. RESV treatment significantly inhibited cell proliferation with or without PDGF-BB stimulation in a dose dependent manner (Fig. 3A). In the cell migration assay, PDGF-BB stimulation significantly promoted cell migration. RESV treatment significantly inhibited cell migration with or without PDGF-BB stimulation in a dose dependent manner (Fig. 3B). Immunofluorescence staining showed prominently increased fibronectin expression 12 h after stimulation with TGF-β2. RPE cells treated by RESV showed less expression of fibronectin stimulated with or without TGF-β2 (Fig. 3C). Trypan blue staining (supplemental Figure 5A), MTT (supplemental Figure 5B) and TUNEL assay (supplemental Figure 5C) revealed no cell toxicity after treatment with RESV at doses of 25, 50 or 100 μM.

### RESV inhibits progression of PVR *in vivo*

**Figure 4:**
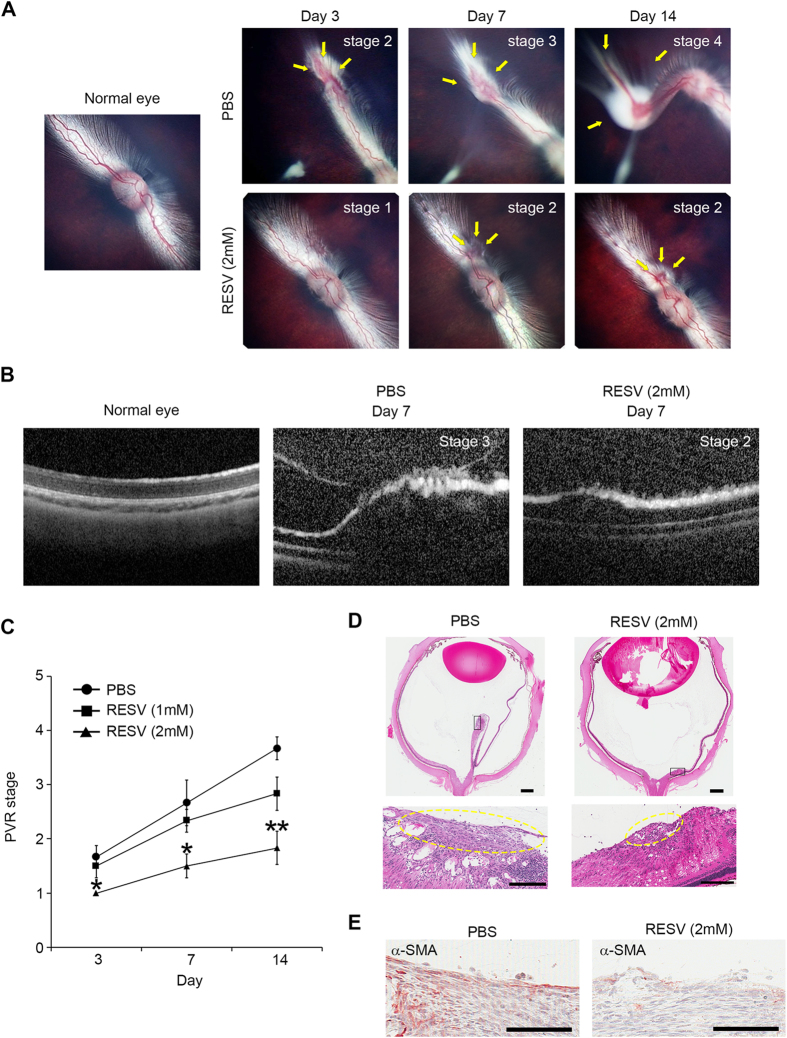
RESV inhibits progression of experimental PVR in rabbits. (**A**) Ocular fundus photographs of normal eye and eyes with PVR after treatment with PBS and RESV at 2 mM. PVR classification was based on the extent of PVR in the lesion (arrows). Details of PVR grading procedures for Grades 1-5 for the rabbit PVR model are described in the Methods section. (**B**) Image of optical coherence tomography taken from normal eye, eyes with PVR after treatment with PBS and RESV at 2 mM. (**C**) Progression of PVR stages in PVR model of rabbits injected with PBS, RESV at 1 mM, and RESV at 2 mM. **P < 0.01. *P < 0.05. Data are presented as mean ± SEM. *n* = 6/group. (D) Section of a whole eye stained with hematoxylin and eosin (H&E) in a rabbit injected with PBS or RESV at 2 mM. Scale bars: 1 mm. Boxed region in the sections is shown at higher magnification. The epiretinal fibrotic membranes are indicated by yellow dotted circles. Scale bars: 200 μm. (E) Immunoreactivity to α-SMA in the fibrotic membranes in the eyes injected with PBS and RESV at 2 mM. Scale bars: 100 μm.

We next investigated whether RESV can inhibit progression of PVR *in vivo* using a rabbit model of PVR^25^. We graded the stages of PVR in rabbit eyes injected with PBS or RESV (1 mM and 2 mM) based on the examination of the fundus images. Endovitreous fibrotic membranes can be seen in the eyes injected with PBS, and PVR progressed up to 14 days post induction. Representative fundus pictures showed focal traction and engorgement (stage 2) on day 3, localized detachment of medullary ray (stage 3) on day 7 and extensive retinal detachment (stage 4) on day 14 (Fig. 4A). Optical coherence tomography (OCT) clearly demonstrated retinal traction with an epiretinal membrane on day 7 post induction (Fig. 4B). By contrast, in the eyes injected with RESV at 2 mM, no endovitreous fibrotic membranes were seen, and the most advanced PVR stage was stage 2, showing focal traction on day 7 post induction (Fig. 4A,B). Overall, PVR progression was significantly suppressed on days 3, 7 and 14 in the eyes injected with RESV at 2 mM compared to the eyes injected with PBS. No significant differences were seen in the PVR progression between the eyes injected with PBS or with RESV at 1 mM (Fig. 4C). Histological findings confirmed the presence of extensive retinal detachment and epiretinal fibrotic membrane in the eyes injected with PBS, while there was no apparent retinal detachment and reduced formation of epiretinal fibrotic membrane in the eyes injected with RESV at 2 mM (Fig. 4D). Immunohistochemical staining showed prominent α-SMA expression in the epiretinal fibrotic membranes of PVR eyes injected with PBS. In contrast, α-SMA expression was less prominent in epiretinal fibrotic membranes of the PVR eyes injected with RESV at 2 mM (Fig. 4E).

## Discussion

This study demonstrates that RESV inhibits EMT of RPE in a SIRT1-dependent fashion, and the effect of RESV can be mediated by SMAD4 deacetylation. We further propose RESV as a potential therapeutic application for PVR by showing its suppressive effect on the fibrotic process in both *in vitro* and *in vivo* models of PVR.

In PVR, EMT of RPE is one of the important steps in the fibrotic process; that is, RPE that undergo EMT acquire a fibroblast phenotype with increased capacity to proliferate and migrate and with the ability to produce ECM, which facilitate the formation of fibrotic membrane^26^,^27^,^28^,^29^. In our *in vitro* culture system, differentiated RPE are induced to undergo EMT by TGF-β. During the EMT process, RPE cells lose the epithelial differentiation markers E-cadherin and ZO-1 and gain the mesenchymal marker α-SMA^30^,^31^,^32^. Since loss of RPE cell-cell contact is the initiating event for EMT, followed by exposure to EMT-driving growth factors that lead to completion of EMT^7^,^29^, this culture system is useful to study an early phase of PVR pathogenesis. Thus, our data show *in vitro* the facilitatory role of RESV in MET and its inhibitory effect on TGF-β2-induced EMT can decrease fibrotic membrane formation, resulting in prevention of PVR progression *in vivo*.

Since RESV allosterically binds to SIRT1 and lowers the Michaelis constant of SIRT1 for acetylated substrate, RESV has been identified as a SIRT1 activator that can regulate key downstream targets through deacetylation^33^,^34^. Our data indicate that RESV activates SIRT1 leading to SMAD4 deacetylation, which modulates the expression of target molecules such as E-cadherin and α-SMA. Our results receive support from a previous report showing SIRT1-induced repression of EMT by SMAD4 deacetylation in kidney tubular epithelial cells and exacerbated injury induced kidney fibrosis^35^.

We demonstrate that SIRT1 has a facilitatory role in MET by showing expression changes of EMT markers, reduced α-SMA and increased E-cadherin after SIRT1 overexpression. However, the SIRT1 overexpression has much less impact on expression of EMT markers than does RESV treatment. Moreover, SIRT1 silencing almost completely abrogates the effect of RESV on MET. These findings imply that activated SIRT1 in the presence of RESV plays a crucial role in RESV-induced MET; whereas inactivated SIRT1 in the absence of RESV has comparatively less capability to regulate MET phenotypes.

The inhibitory effect of RESV on RPE EMT can presumably lead to an anti-fibrotic response, such as anti-proliferation and reduced migration and fibronectin synthesis. Nevertheless, it could significantly inhibit PDGF-BB-induced cell migration. These findings indicate that its inhibitory effect can be mediated by alteration of the signaling pathway related to PDGF-BB-induced migration but not through EMT inhibition. Consistent with our finding, Chan *et al.*^36^ reported that RESV inhibited PDGF-BB-induced migration of human RPE cell line ARPE19. They reported that its inhibitory effect is mediated by suppression of PDGF receptor β, phosphatidylinositol-3 kinase/Akt pathway activation and mitogen-activated protein kinase activation^36^. RESV is a potent inhibitor of multiple signaling pathways related to fibrosis development, e.g., TGF-β/SMAD, NF-κB signaling and ERK signaling pathways^19^,^37^,^38^. Resveratrol has also been shown to impact several transcription factors (AP-1, Egr-1 and NF-κB), and molecular components involving the cell cycle (p21^Cip1/WAF1^), growth/apoptosis (p53, Bcl-2, Bax, Survivin)^39^,^40^. Recently, we found that in RPE, RESV suppresses hypoxia inducible factor-1α accumulation, and vascular endothelial growth factor (VEGF) secretion, while in endothelial cells it inhibits VEGF-R2 phosphorylation, suggesting a role for RESV in the inhibition of angiogenesis^41^.

In conclusion, our data show that RESV inhibits EMT of RPE and fibrosis associated with PVR. The present work suggests the potential use of RESV as a therapeutic agent to prevent the progression of PVR.

## Methods

### RPE Cell Culture

Human RPE cells were isolated from postmortem fetal eyes (gestational age 16–18 weeks) from Advanced Bioscience Resources, Inc. (Alameda, CA, USA). Primary cultures of RPE cells were established as described previously^42^. Cells were cultured in DMEM with 2 mM l-glutamine, 100 U/mL penicillin, 100 μg/mL streptomycin, and 10% heat-inactivated fetal bovine serum (FBS). The experiments were performed using cultured human RPE cells at two to four passages that were grown until they were just confluent with hexagonal shape and without visible pigmentation. In our experience, human RPE cells develop pigmentation only when they are cultured at confluence for an extended period of time (several weeks). RPE cells were starved overnight with serum free media and replaced with DMEM containing 3% FBS. After 1 h pretreatment with RESV, the cells were stimulated with recombinant TGF-β2 (10 ng/ml, Sigma-Aldrich, St. Louis, MO, USA) for up to 2 days.

### Transfection

RPE cells were cultured in a 12-well plate to a confluence of 50–70%. SiRNA targeting SIRT1 or control siRNA (Santa Cruz Biotechnology, Inc., Dallas, TX, USA) were mixed (10 nM at final concentration) with 1 μL reagent (Lipofectamine® RNAiMAX; Life Technologies, Grand Island, NY, USA) in 100 μL medium (Opti-MEM; Life Technologies). To overexpress SIRT1, 1 μg of pCMV6-XL4 plasmid encoding human SIRT1 (OriGene Technologies, Rockville, MD, USA) or empty vector was mixed with 3 μL reagent (Lipofectamine® LTX; Life Technologies) in 100 μL medium (Opti-MEM; Life Technologies). The mixture was added to the RPE cells and incubated for 24 h. The composite transfection mixture was removed and replaced with DMEM containing 3% FBS.

### RNA isolation and real-time RT-PCR (RT-qPCR)

Total RNA was isolated using RNeasy kit (QIAGEN, Valencia, CA, USA) according to the manufacturer’s instructions. Real-time RT-PCR was performed, as described previously^43^. Gene expression levels were normalized relative to GAPDH mRNA and β-actin mRNA and reported as fold change over controls. Analyses using β-actin mRNA are shown only in supplementary figures. The primer sequences used are as follows: E-cadherin, 5′-ATTTTTCCCTCGACACCCGAT-3′ and 5′-TCCCAGGCGTAGACCAAGA-3′; α-SMA, 5′-TCTGTAAGGCCGGCTTTGC-3′ and 5′-TGTCCCATTCCCACCATCA-3′; ZO-1, 5′-GGACTCTCGCTG GTCTACCTRGGGCACAATATGCAGGCAGA-3′; SIRT1, 5′-TCCTGGACAATTCCAGCCATCTCT-3′ and 5′-TTCCAGCGTGTCTATGTTCTGGGT-3′; GAPDH, 5′-ACAGTCGCCGCATCTTCTT-3′ and 5′-CTTGATTTTGGAGGGATCTCGC-3′; and β-actin, 5'-CATGTACGTTGCTATCCAGGC-3' and 5'-CTCCTTAATGTCACGCACGAT-3'.

### Western blotting

Western blotting was performed as described previously^44^. Total cell lysates were extracted with lysis buffer. The extracted cell lysates were subjected to Tris-HCl polyacrylamide gels, and the blots were incubated with antibodies (Abs, Supplementary Table). Visualization was performed using an enhanced chemiluminiscence (Amersham Pharmacia Biotech, Cleveland, OH, USA) detection system. GAPDH was used as the loading control.

### Rabbit PVR model

PVR was induced in the right eye of adult pigmented rabbits, each weighing 2.5 to 3.5 kg, following a reported protocol^25^. Briefly, after 0.2 ml of vitreous was removed using a 25-gauge needle, 100 ul PBS with or without RESV containing rabbit RPE cells (passage 2–3, 15 × 10^4^) and 50 ng PDGF-BB (R&D Systems, Minneapolis, MN, USA) were injected into the vitreous cavity 3 mm posterior to the corneal limbus. The eyes were injected with PBS or RESV in each group on day 3. Clinical observations with digital fundus camera (RetCam II; Clarity Medical Systems, Inc., Pleasanton, CA, USA) and OCT (Heidelberg Engineering, Inc., Heidelberg, Germany) were performed for as long as 14 days after injection, and PVR was classified according to Fastenberg *et al.*^45^. In brief, the stage of PVR grades was based on the findings of retinal fundus examination as following: stage 1, intravitreal membrane; stage 2, focal traction; localized vascular changes; hyperemia; engorgement; blood vessel elevation; stage 3, localized detachment of medullary ray; stage 4, extensive retinal detachment; total medullary ray detachment; peripapillary retinal detachment; stage 5, total retinal detachment; retinal folds and holes. The eyes were enucleated on day 14 for histologic analysis.

### Immunofluorescence staining and immunohistochemistry

RPE cells were cultured in 4-well multi-chamber slides (Thermo Scientific, Waltham, MA, USA). The cells were rinsed in PBS for 3 min and fixed and permeabilized with ice-cold methanol for 20 min. After a 30-min blocking step with 10% goat serum, the cells were incubated with primary Abs (Supplementary Table) for 24 h at 4 °C and then incubated with secondary Abs. The specimens were mounted in mounting medium including DAPI (Vector Laboratories) and examined with a Perkin Elmer spinning disk confocal microscope. Rabbit eyes were embedded in paraffin and cut at a 5 μm thickness. Immunohistochemistry was performed as described previously^44^. The retinal sections were treated with 0.3% hydrogen peroxide for 10 min to block endogenous peroxide. The sections were then blocked in 10% normal goat serum for 30 min and incubated with Abs (Supplementary Table) overnight at 4 °C. Slides were washed with PBS and incubated with biotinylated IgG (Vector Laboratories) for 1 h. Immunoperoxidase was detected with aminoethylcarbazole (AEC) as the chromogen, using AEC kit (Life Technologies) according to the manufacturer’s protocol.

### Immunoprecipitation

Five hundred micrograms of total retinal proteins were incubated in immunoprecipitation lysis buffer (Thermo Scientific) containing primary anti-SMAD4 antibody overnight at 4 °C with gentle shaking. The immune complexes were collected with the use of anti–rabbit agarose beads for 2 hours and centrifuged at 14000 g for 30 seconds. After washing with lysis buffer, Laemmli sample buffer (Bio-Rad Laboratories Inc, Irvine, CA, USA) was added to the residual agarose with immune complexes and heated at 95 °C for 3 minutes. The samples were loaded onto Tris-HCl polyacrylamide gels and western blotting was performed by the use of anti-acetylated lysine Ab (Supplementary Table).

### Cell proliferation assay

Cell proliferation in RPE cells was analyzed using bromodeoxyuridine (BrdU)-ELISA (Roche Applied Science, Indianapolis, IN, USA) according to the manufacturer’s instructions with a 2 h BrdU incubation with or without RESV.

### Cell migration assay

Cell migration assay was performed using an Oris 96-well cell migration assay kit (Platypus Technologies, Madison, WI, USA) according to the manufacturer’s instructions as described previously^44^. Briefly, 5 × 10^4^ cells were seeded in each well. After 1 h pretreatment with DMEM with 3% FBS containing RESV and recombinant PDGF-BB at 50 ng/ml with 5 μM aphidicolin (Sigma-Aldrich) to inhibit cell division, the stoppers were removed to allow cells to migrate into the detection zone. The cells were incubated for 48 h and stained with PBS containing calcein AM (Life Technologies) for 1 h. The area of cell migration was determined using Photoshop software (Adobe Systems, San Jose, CA, USA).

### Trypan blue exclusion assay

Cell viability was determined by trypan blue exclusion assay. After the cells were detached with trypsin-EDTA, the pellet was resuspended in PBS and the number of viable cells was assessed in a Neubauer’s cell chamber, using trypan blue as non-viability stain.

### MTT assay

Cell viability was assessed using a 3-(4,5-dimethylthiazol-2-yl)-2,5-diphenyl tetrazolium bromide (MTT) assay according to the manufacturer’s instructions (Life Technologies).

### TUNEL

Apoptosis was detected by the terminal deoxynucleotidyl transferase (TdT)-mediated dUTP-biotin nick end-labeling (TUNEL) method according to the manufacturer’s instructions (Roche Applied Science).

### Statistical analyses

All results are expressed as means ± SEM. The statistical significance of differences between groups was analyzed using the Tukey HSD test or two-tailed Student’s *t-*test. In comparison with the control group, Dunnett’s t test was applied. We performed one-way ANOVA, with a Bonferroni correction for multiple comparisons where applicable. Differences were considered significant at *P* < 0.05. Statistical analyses were performed using JMP^®^ version 7.0.1 (SAS Institute, Cary, NC, USA).

### Study approval

All procedures used in these studies were conducted in accordance with applicable regulatory guidelines at the University of Southern California, principles of human research subject protection in the Declaration of Helsinki and principles of animal research in the Association for Research in Vision and Ophthalmology Statement for the Use of Animals in Ophthalmic and Vision Research. Human postmortem fetal eyes were obtained from Advanced Bioscience Resources Inc., under USC Health Sciences Institutional Review Board (IRB) protocol HS-947005, and written informed consent was obtained by Advanced Bioscience Resources Inc. from all donors. All vertebrate animal research was approved by the Keck School of Medicine Institutional Animal Care and Use Committee (IACUC) protocol HS-947005.

## Additional Information

**How to cite this article**: Ishikawa, K. *et al.* Resveratrol inhibits epithelial-mesenchymal transition of retinal pigment epithelium and development of proliferative vitreoretinopathy. *Sci. Rep.* **5**, 16386; doi: 10.1038/srep16386 (2015).

## Supplementary Material

Supplementary Information
