# Supplementary material for: Resveratrol inhibits epithelial-mesenchymal transition of retinal pigment epithelium and development of proliferative vitreoretinopathy
Source: Sci Rep. 2015 Nov 10;5:16386. doi: 10.1038/srep16386 (PMC4639835; doi:10.1038/srep16386)
Supplement: Supplementary Information [file srep16386-s1.pdf]

Resveratrol inhibits epithelial-mesenchymal transition of retinal pigment epithelium and development of proliferative vitreoretinopathy

Keijiro Ishikawa,[1,2] Shikun He,[2, 3] Hiroto Terasaki,[1] Hossein Nazari,[2] Huiming Zhang,[2] Christine Spee,[2] Ram Kannan,[1] and David R Hinton[2,3*]

Arnold and Mabel Beckman Macular Research Center, Doheny Eye Institute[1], Los Angeles, CA, USA;

Departments of Ophthalmology[2] and Pathology[3], Keck School of Medicine of the University of Southern California, Los Angeles, CA, USA

*Corresponding author

Supplementary Material

Supplementary information contains: Supplementary Table, Supplementary Figures and Legends.

Supplementary Table

Antibodies

Target molecule	Antibody type	Application	Source
α-SMA	mouse monoclonal	WB, IHC	Sigma-Aldrich
E-cadherin (24E10)	rabbit monoclonal	WB, IHC	Cell Signaling
ZO-1	rabbit monoclonal	WB	Cell Signaling
SMAD4	rabbit polyclonal	WB, IP	Santa Cruz Biotech
Acetylated-Lysine	rabbit polyclonal	WB	Cell Signaling
Sirtuin 1	rabbit polyclonal	WB	Santa Cruz Biotech
Fibronectin	mouse monoclonal	IHC	R&D Systems
GAPDH	mouse monoclonal	WB	Millipore

α-SMA = α-smooth muscle actin; WB = western blotting; IHC = immunohistochemistry;

IP = immunoprecipitation.

Supplementary Figures and Legends

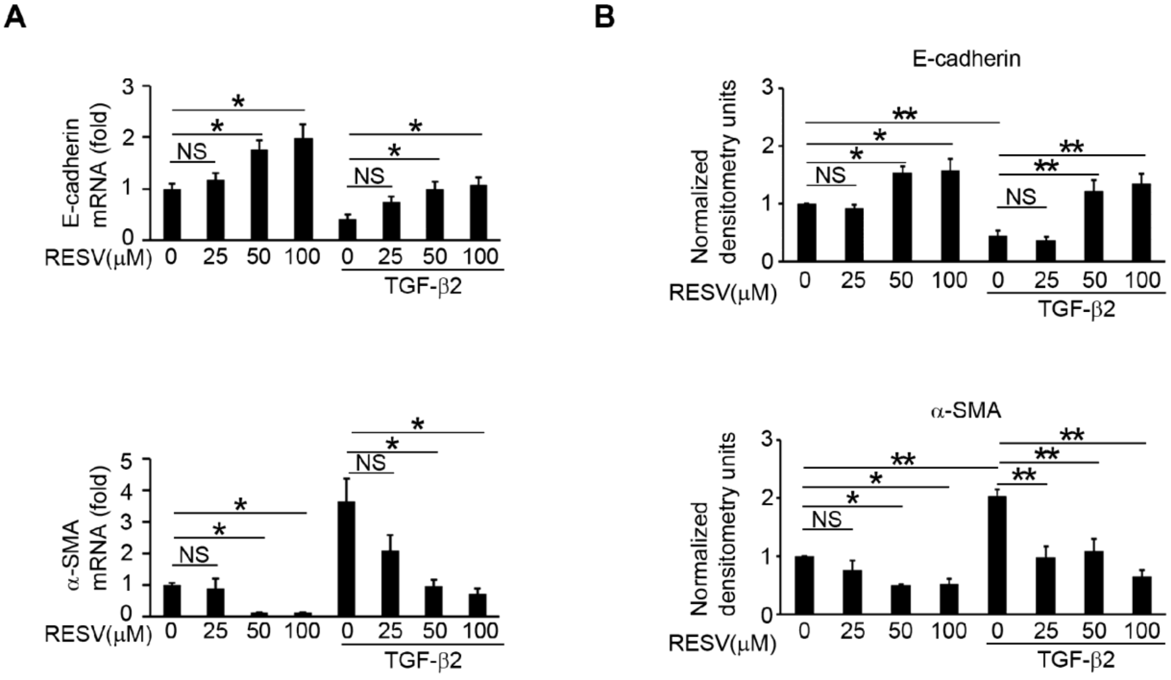

Supplementary Fig 1

RESV induces MET and inhibits TGF-β2 induced EMT of RPE cells. (A) mRNA expression of E-cadherin and α-SMA are shown as relative fold to control normalized to β-Actin. NS, not significant. *, $P<0.05$. Data are presented as mean ±SEM. $n = 4$/group. (B) Western blot analysis of protein expression of E-cadherin and α-SMA quantified by densitometry are shown as relative fold to control normalized to GAPDH. NS, not significant. * *, $P<0.01$. *, $P<0.05$. Data are presented as mean ±SEM. $n = 3$/group.

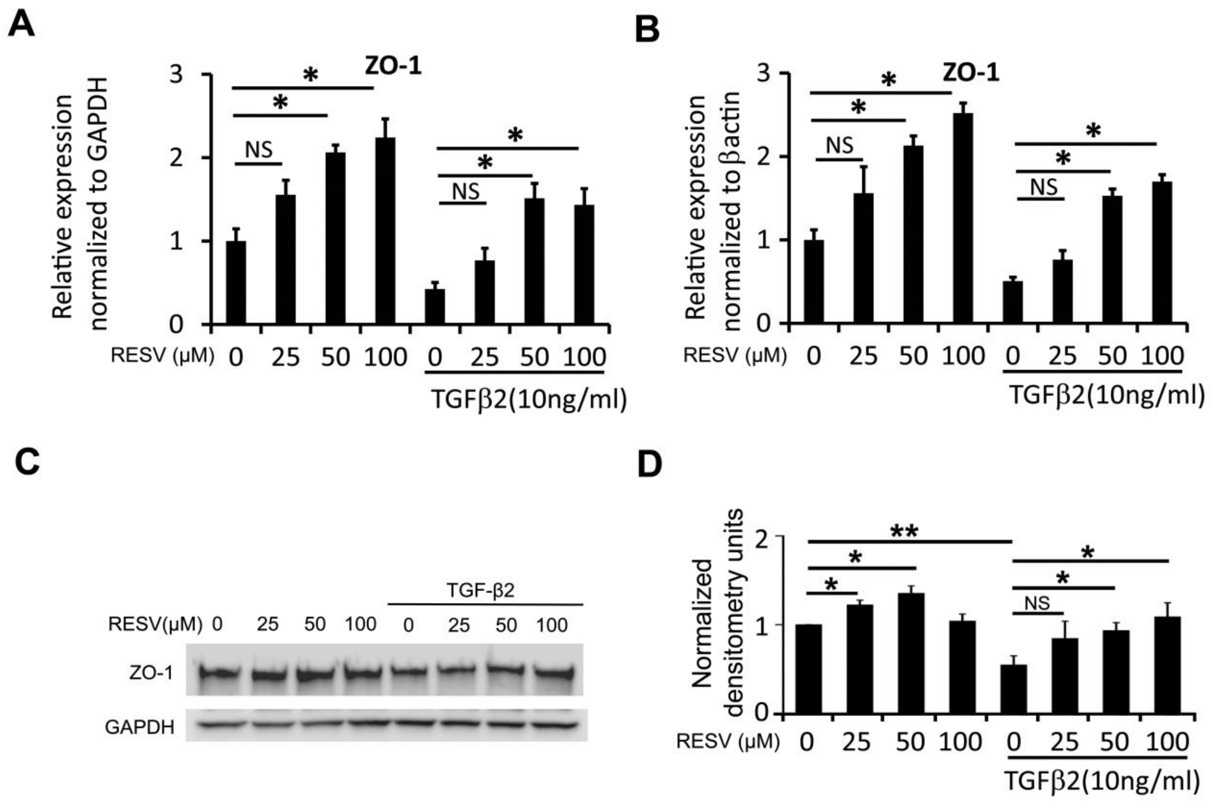

Supplmentary Fig 2.

TGFβ2-induced suppression of ZO-1 gene and protein expression and dose-dependent upregulation with RESV in RPE cells. ZO-1 gene expression (normalized to GAPDH and β-actin) (A,B) and protein expression (C,D) after TGFβ2 treatment (10 ng/ml, 48h) with or without RESV (0-100 μM) are shown. NS, not significant. * *, *P*<0.01. *, *P*<0.05. Data are presented as mean ±SEM. *n* = 4/group.

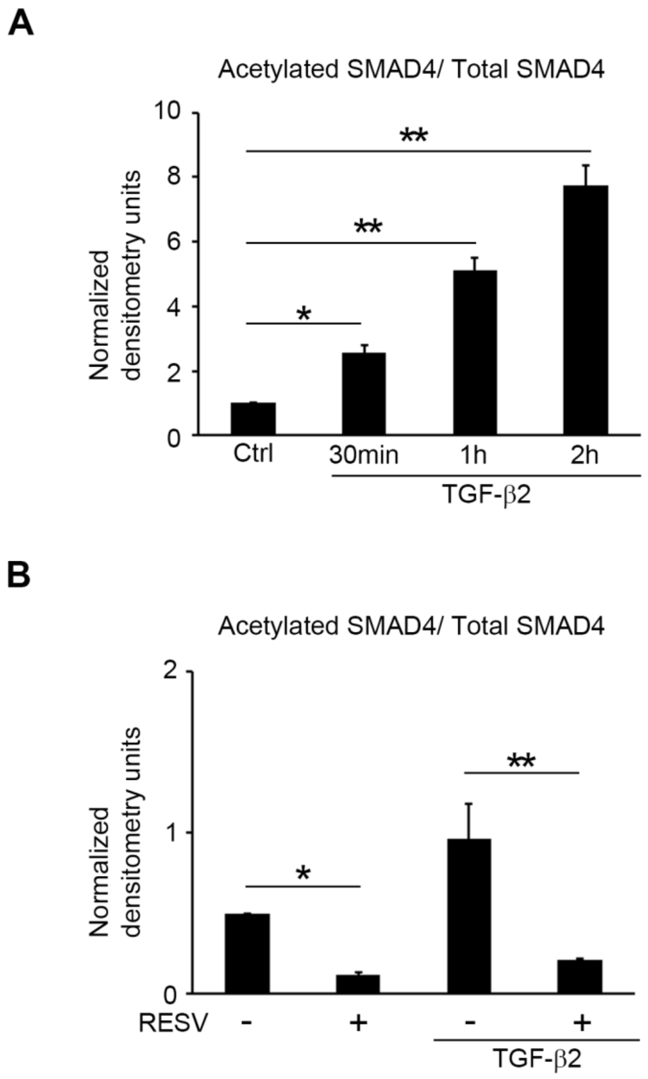

Supplementary Fig 3.

TGF-β2 induces acetylation of SMAD4. RESV inhibits acetylation of SMAD4 with or without TGF-β2 stimulation. Protein expression of acetylated SMAD4 quantified by densitometry is shown as relative fold to control normalized to total SMAD4. (A) Quantification of data shown in Figure 1D. (B) Quantitation of data shown in Figure 1E. * *, $P<0.01$. *, $P<0.05$. Data are presented as mean ±SEM. $n = 3$/group.

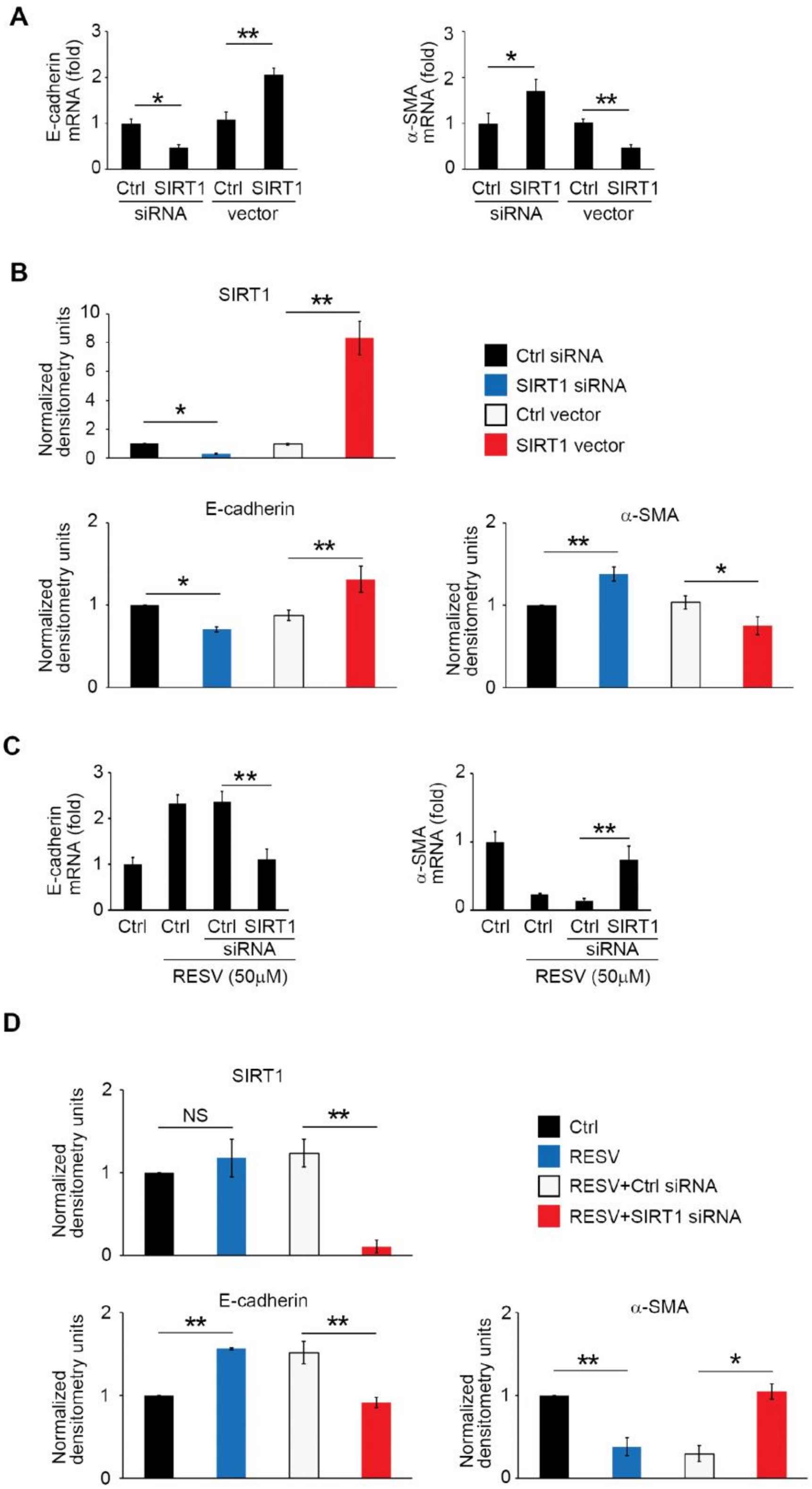
A
E-cadherin mRNA (fold)
Ctrl SIRT1 Ctrl SIRT1
siRNA vector
α-SMA mRNA (fold)
B
SIRT1
Normalized densitometry units
Ctrl siRNA
SIRT1 siRNA
Ctrl vector
SIRT1 vector
E-cadherin
α-SMA
C
Ctrl Ctrl Ctrl SIRT1
siRNA
RESV (50μM)
D
NS
Ctrl
RESV
RESV+Ctrl siRNA
RESV+SIRT1 siRNA

Supplementary Fig 4

Facilitatory role of RESV on MET is dependent on SIRT1. (A and C) mRNA expression of E-cadherin and α-SMA are shown as relative fold to control normalized to β-Actin. * *, $P<0.01$. *, $P<0.05$. Data are presented as mean ±SEM. $n = 4$/group. (B and D) Protein expression of SIRT1, E-cadherin and α-SMA quantified by densitometry is shown as relative fold to control normalized to GAPDH. (B) Quantification of data shown in Figure 2B are from 3 independent experiments. (D) Quantification of data shown in Figure 2D are from 3 independent experiments. NS, not significant. * *, $P<0.01$. *, $P<0.05$. Data are presented as mean ±SEM. $n = 3$/group.

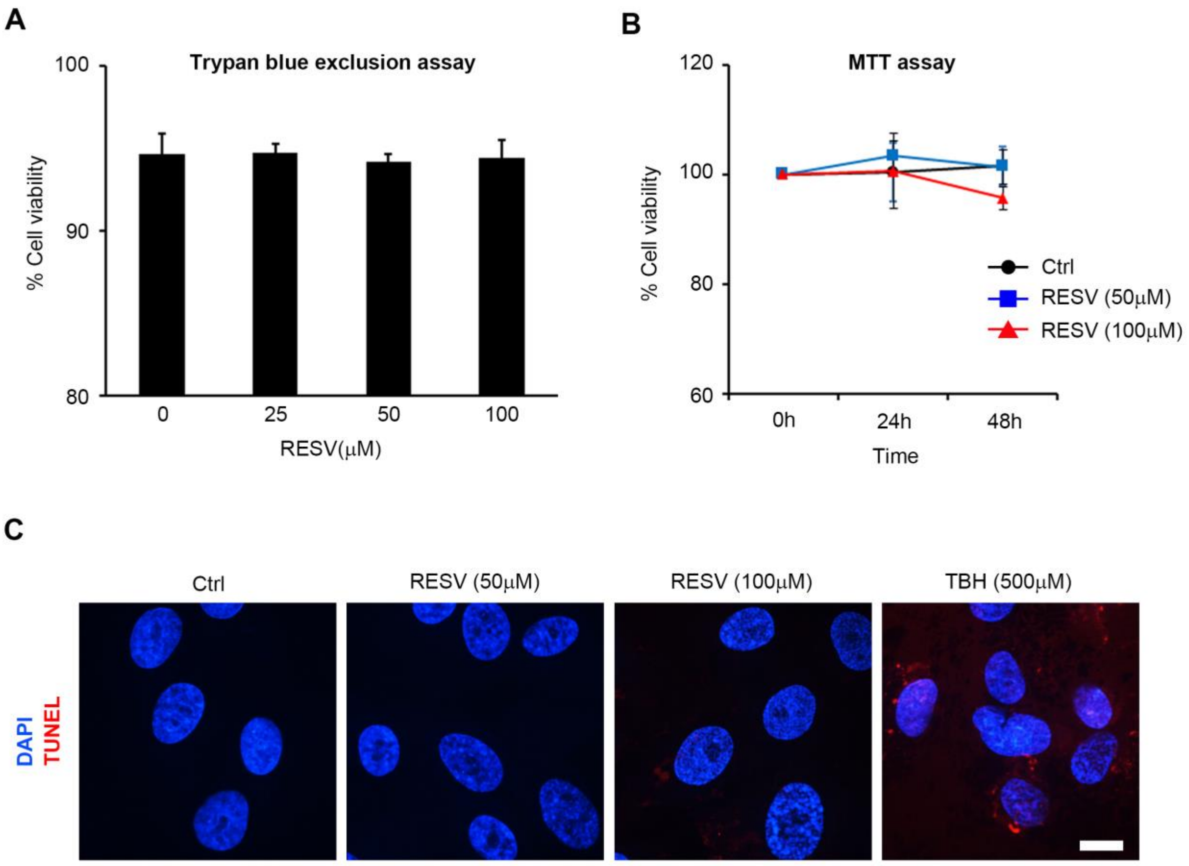

Supplementary Fig 5.

RESV does not cause toxicity at the indicated doses in RPE cells. (A) Percentage of viable cells treated with RESV for 48 h were assessed by trypan blue exclusion assay. *n* = 4/group. (B) Cell viability was assessed by MTT assay after treatment with RESV for 24 h and 48 h. Values are expressed as a percentage relative to the untreated control. *n* = 4/group. (C) TUNEL staining (red) with cells after treatment with RESV for 48 h. Nuclei are stained blue. As a TUNEL positive control, RPE cells treated with TBH (t-butyl hydroperoxide at 500 μM) for 4 h were used. Scale bar: 10 μm.

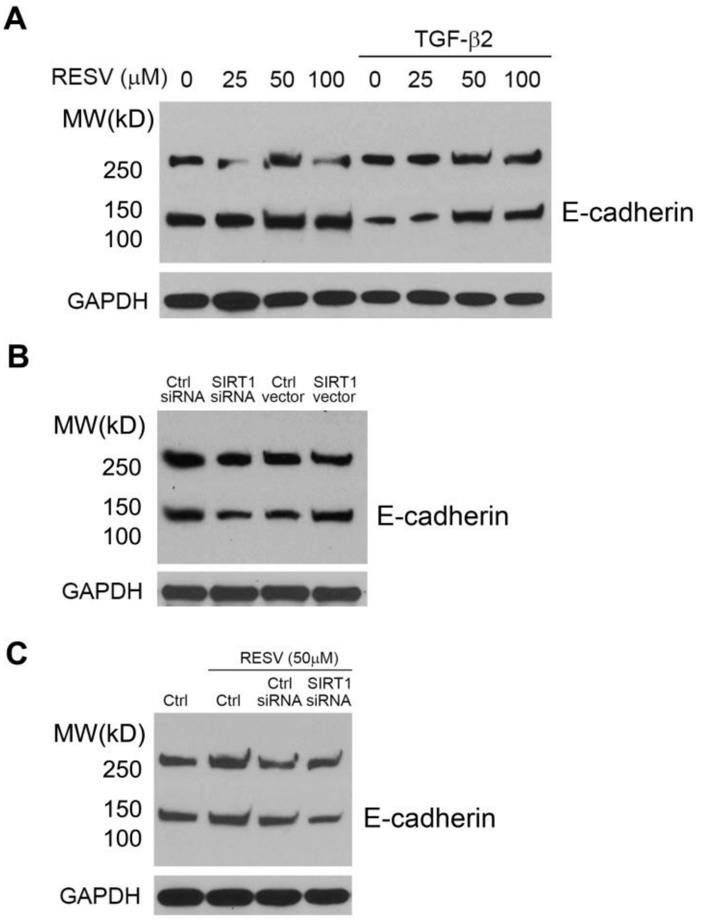

Supplementary Fig 6. RESV induces MET in RPE cells and the facilitatory role of SIRT1 in MET induction. Whole gels for western blot analysis of E-cadherin expression showing a band with a molecular size of 135 kD along with the house keeping gene GAPDH are shown. The gel also shows a second uncharacterized band around 270 kD for E-cadherin. Panels A, B and C in this supplemental figure represent whole gel images for the original figures 1B, 2B and 2D respectively.
